# Area-level risk factors for adverse birth outcomes: trends in urban and rural settings

**DOI:** 10.1186/1471-2393-13-129

**Published:** 2013-06-10

**Authors:** Shia T Kent, Leslie A McClure, Ben F Zaitchik, Julia M Gohlke

**Affiliations:** 1 Department of Environmental Health Sciences, University of Alabama at Birmingham (UAB), Ryals Public Health Building 530, 1665 University Ave, Birmingham, AL, 35294, USA; 2 Department of Biostatistics, University of Alabama at Birmingham (UAB), Ryals Public Health Building 327, 1665 University Ave, Birmingham, AL, 35294, USA; 3 Department of Earth and Planetary Sciences, Johns Hopkins University, 327 Olin Hal 3400 N. Charles Street, Baltimore, MD, 21218, USA

**Keywords:** Health status disparities, Low birth weight, Premature birth, Rural health, Urban health

## Abstract

**Background:**

Significant and persistent racial and income disparities in birth outcomes exist in the US. The analyses in this manuscript examine whether adverse birth outcome time trends and associations between area-level variables and adverse birth outcomes differ by urban–rural status.

**Methods:**

Alabama births records were merged with ZIP code-level census measures of race, poverty, and rurality. B-splines were used to determine long-term preterm birth (PTB) and low birth weight (LBW) trends by rurality. Logistic regression models were used to examine differences in the relationships between ZIP code-level percent poverty or percent African-American with either PTB or LBW. Interactions with rurality were examined.

**Results:**

Population dense areas had higher adverse birth outcome rates compared to other regions. For LBW, the disparity between population dense and other regions increased during the 1991–2005 time period, and the magnitude of the disparity was maintained through 2010. Overall PTB and LBW rates have decreased since 2006, except within isolated rural regions. The addition of individual-level socioeconomic or race risk factors greatly attenuated these geographical disparities, but isolated rural regions maintained increased odds of adverse birth outcomes. ZIP code-level percent poverty and percent African American both had significant relationships with adverse birth outcomes. Poverty associations remained significant in the most population-dense regions when models were adjusted for individual-level risk factors.

**Conclusions:**

Population dense urban areas have heightened rates of adverse birth outcomes. High-poverty African American areas have higher odds of adverse birth outcomes in urban versus rural regions. These results suggest there are urban-specific social or environmental factors increasing risk for adverse birth outcomes in underserved communities. On the other hand, trends in PTBs and LBWs suggest interventions that have decreased adverse birth outcomes elsewhere may not be reaching isolated rural areas.

## Background

Significant racial and income disparities in birth outcomes exist in the US [1] and have persisted or even widened, despite a concerted research and practice effort aimed at reducing these disparities [2]. Previous research has shown that low-income individuals have higher preterm birth (PTB) and low birth weight (LBW) rates [3-7]. In addition, non-Hispanic African Americans compared to Non-Hispanic whites consistently have had at least twice the rate of LBW babies and 1.5 times the rate of PTBs [8], and previous studies controlling for socioeconomic status (SES) factors show racial disparities persist [9-11].

Health disparities across the urban–rural gradient also exist; both inner city and rural residents, compared to suburban, have poorer health outcomes [3]. The trends in urban–rural health disparities may be affected by demographic transitions, such that some rural areas have become more isolated as more people move into urban areas. Several explanations for urban–rural disparities in adverse birth outcomes have been suggested including higher prevalence of smoking, health care inequalities, and increased exposure to environmental hazards [12-16]. Taken together, these results suggest low socioeconomic status (SES) African American individuals in inner city and rural areas may be particularly vulnerable to adverse birth outcomes.

Previous research has not determined the extent that area-level socioeconomic factors contribute to birth outcome disparities or whether rurality modifies this effect. Several studies have found significant area-level socioeconomic adverse birth outcome disparities remained after individual variables were included in the model [14,17-19]. However, some studies found that individual-level factors generally had stronger relationships with birth outcomes than area-level variables; and have also found that after accounting for individual risk factors that area-level poverty and other race factors were attenuated, and in some demographic strata did not have significant relationships with some adverse birth outcomes [17,20,21]. If there are specific area-level factors that play a role in determining individual-level risk, one might expect modification of the effect across urban–rural geographies, since several area-level environmental (e.g. air pollution) and social (e.g. isolation) risk factors are divergent across urban- rural settings.

Unlike the rest of the US, both urban and rural areas in the Deep South have significant non-Hispanic African American populations, allowing for characterization of racial health disparities across the urban–rural gradient. Alabama is consistently among the states with the highest PTB and LBW rates in the US. In 2010, the PTB rate in Alabama was 15.6% compared to the national average of 12.0%, and Alabama’s African American communities have particularly high PTB and LBW rates [22].

Here we examine whether contributions of individual or area-level factors to health disparities in the Deep South vary by urban–rural status over the last 20 years. We have two objectives: (1) determine whether 20-year temporal trends in PTB and LBW in Alabama vary by rurality and (2) determine if rurality modifies the effects of area-based racial composition or poverty with regards to individual-level risk for PTB and LBW.

## Methods

### Birth records

Alabama birth records for 1990–2010 warm season months (May-September) obtained from the Alabama Department of Public Health (ADPH) were available for these analyses. Study protocol was reviewed and approved through the ADPH and University of Alabama at Birmingham Institutional Review Boards. Births were classified as PTB (<37 weeks) and LBW (<2,500 grams). Births before 24 weeks or less than 200 grams were excluded from analyses [23,24]. Births with missing variables, or variable values which were not included as a variable category were excluded from all analyses. Of the 506,056 births recorded, 490, 366 (96.9%) with full covariate data were available for analyses.

### ZIP code-level measures

**Figure 1:**
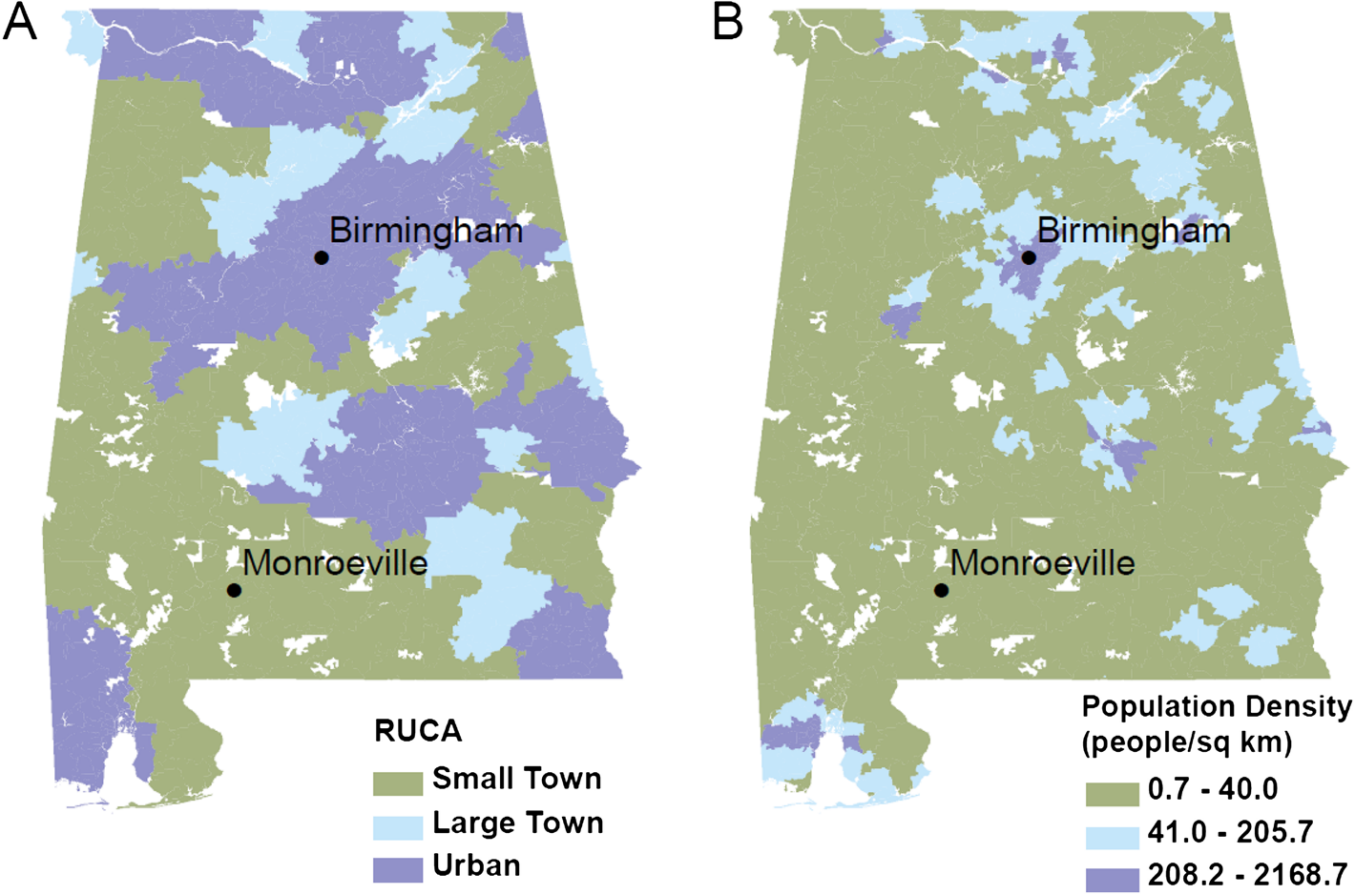
Spatial Distributions of (A) Rural–urban Commuting Area Code categories and (B) Population Density Tertiles Across Alabama.

We merged birth records with Census 2000 ZIP code-level percentages of poverty and self-identified non-Hispanic African American. As has been previously done, we allowed for non-linearity by classifying these variables into quartiles [19]. Since there is no single accepted definition of rurality, and different rurality measures correlate with different exposures, we merged birth records with two common ZIP code-level measures of rurality that explicitly identify isolated rural areas (Rural–urban Commuting Area Codes (RUCAs) version 2.0) and highly urbanized areas (Census 2000 population densities calculated from land surface areas and total populations) (Figure 1). We classified RUCAs using the suggested “categorization B”, which divides ZIP codes into “urban focused”, “large rural city/town (micropolitan) focused”, and “small rural and isolated town focused” categories. We classified Census 2000 population densities into tertiles.

### Statistical analyses

To examine long-term yearly time trends in PTB and LBW by category of rurality, we developed logistic regression models both with and without B-splines. Since preliminary models indicated that overall PTB rates peaked in 2005 and LBW rates peaked in 2006, we tested whether linear changes in PTB or LBW rates before and after these respective peaks differed by rurality category using models with year*rurality interaction terms. Separate models used B-splines with odds ratios (ORs) and 95% confidence intervals (CIs) comparing the different rurality categories at three different time points on the splines to determine differences in the likelihoods of PTB and LBW. The three time points selected for each outcome correspond to the first (1991) and last (2010) years in the study, as well as the year corresponding to peak PTB or LBW rates. Crude models were run, as well as models including the individual-level variables of parity (first or subsequent birth), payment method (Medicaid, private insurance, or self-payment), years of education (less than 12, 12, 13–15, or 16 or more), race (non-Hispanic African American, non-Hispanic white, or Hispanic) and a natural spline for mother’s age.

To examine relationships between adverse birth outcomes and individual and ZIP code-level poverty and race, mixed-effects logistic regression models were run, which included a random ZIP code intercept to estimate area-level variation [17,19,25]. All models in this part of the analysis included a B-spline for year. Final models also included the individual-level variables of parity, payment method, education, race, and a natural spline for mother’s age. A term in each model was included to test for multiplicative interaction between area-level poverty or race and rurality.

Statistical analyses and figures were produced in SAS v9.3 (SAS Institute; Cary, North Carolina) and ArcMap 10.0 (ESRI; Redlands, California).

## Results

### Descriptive statistics

Table 1 shows PTB and LBW rates by individual and area-level characteristics. While PTB and LBW babies are often coincident, more than 40% of PTB babies are not LBW, and 30% of LBW babies are full-term. PTB and LBW rates are higher in mothers who self-paid, were African American, and did not complete college.

**Table 1:** **1991**–**2010 Alabama adverse birth outcomes by individual and ZIP code**-**level characteristics (n = 490,366)**

**Individual-level characteristics**	**PTB, n (%)**	**LBW, n (%)**
Total births	56838 (11.6%)	46606 (9.5%)
PTB		32628 (57.4%)
LBW	32628 (70.0%)	
Payment method		
Medicaid	29795 (12.7%)	27062 (11.5%)
Self-payment	2297 (15.8%)	1902 (13.1%)
Private insurance	24746 (10.3%)	17642 (7.3%)
Race		
African American	23018 (14.7%)	21854 (13.9%)
White	32046 (10.3%)	23478 (7.5%)
Hispanic	1774 (8.7%)	1274 (6.3%)
Years of education		
Fewer than 12	34089 (12.4%)	29857 (10.8%)
12	19326 (11.9%)	16412 (10.1%)
13-15	13096 (11.3%)	10037 (8.6%)
16 or more	9653 (17.0%)	6712 (6.8%)
Parity		
First birth	22370 (10.9%)	19883 (9.7%)
Second or later birth	34468 (12.1%)	26723 (9.4%)
ZIP code-level characteristics		
Percent poverty, n (%)		
1^st^ quartile (0% to 10.3%)	12781 (10.5%)	9255 (7.6%)
2^nd^ quartile (10.4% to 15.1%)	13952 (11.2%)	11043 (8.9%)
3^rd^ quartile (15.2% to 20.8%)	13746 (11.3%)	11576 (9.6%)
4^th^ quartile (20.9% to 70.5%)	16359 (13.3%)	14732 (12.0%)
Percent African American, n (%)		
1^st^ quartile (0% to 8.4%)	12726 (10.5%)	9527 (7.8%)
2^nd^ quartile (8.5% to 21.5%)	12923 (10.5%)	10056 (8.2%)
3^rd^ quartile (21.8% to 44.9%)	14255 (11.6%)	11862 (9.7%)
4^th^ quartile (45.3% to 97.6%)	16934 (13.8%)	15161 (12.4%)
Population density, n (%)		
1^st^ tertile (0.7 to 39.1 people/km2)	18426 (11.3%)	15416 (9.4%)
2^nd^ tertile (39.4 to 197.8 people/km2)	17723 (10.8%)	14043 (8.6%)
3^rd^ tertile (205.7 to 2168.7 people/km2)	20689 (12.7%)	17147 (10.5%)
RUCA, n (%)		
Isolated and small town	9454 (11.5%)	8057 (9.8%)
Large town	6669 (10.8%)	5665 (9.2%)
Urban	40715 (11.8%)	32884 (9.5%)

Abbreviations: *PTB* preterm birth, *LBW* low birth weight, *RUCA* Rural–urban Commuting Area Codes.

Maps of ZIP code-level characteristics show that percent poverty and African American distributions follow similar spatial trends across Alabama (see Additional file 1: Figure S1). LBW rates appear to be higher in the high percent poverty and African American regions in southwestern and Birmingham regions, but not the high percent poverty and low percent African American northern Alabama regions (see Additional file 1: Figure S1). PTB rates follow similar patterns as LBW rates in the Birmingham and central Alabama region, but there are differences in western Alabama, where a larger proportion of ZIP codes have higher LBW, but not higher PTB rates (see Additional file 1: Figure S1). This suggests percent in poverty and percent African American may independently predict birth outcomes, although the high collinearity between these variables makes the relationships difficult to tease apart.

### Rurality spatial distributions

Figure 1 shows the divergent spatial distributions of rurality across two commonly used rural–urban classification systems. RUCA-defined rurality identifies isolated rural areas and shows the largest spatially contiguous regions of rurality categories, since commuting patterns play a primary role in the definition (Figure 1A), whereas Census 2000 population density tertiles (Figure 1B) more clearly define highly urbanized areas.

### PTB and LBW time trends by rurality

**Figure 2:**
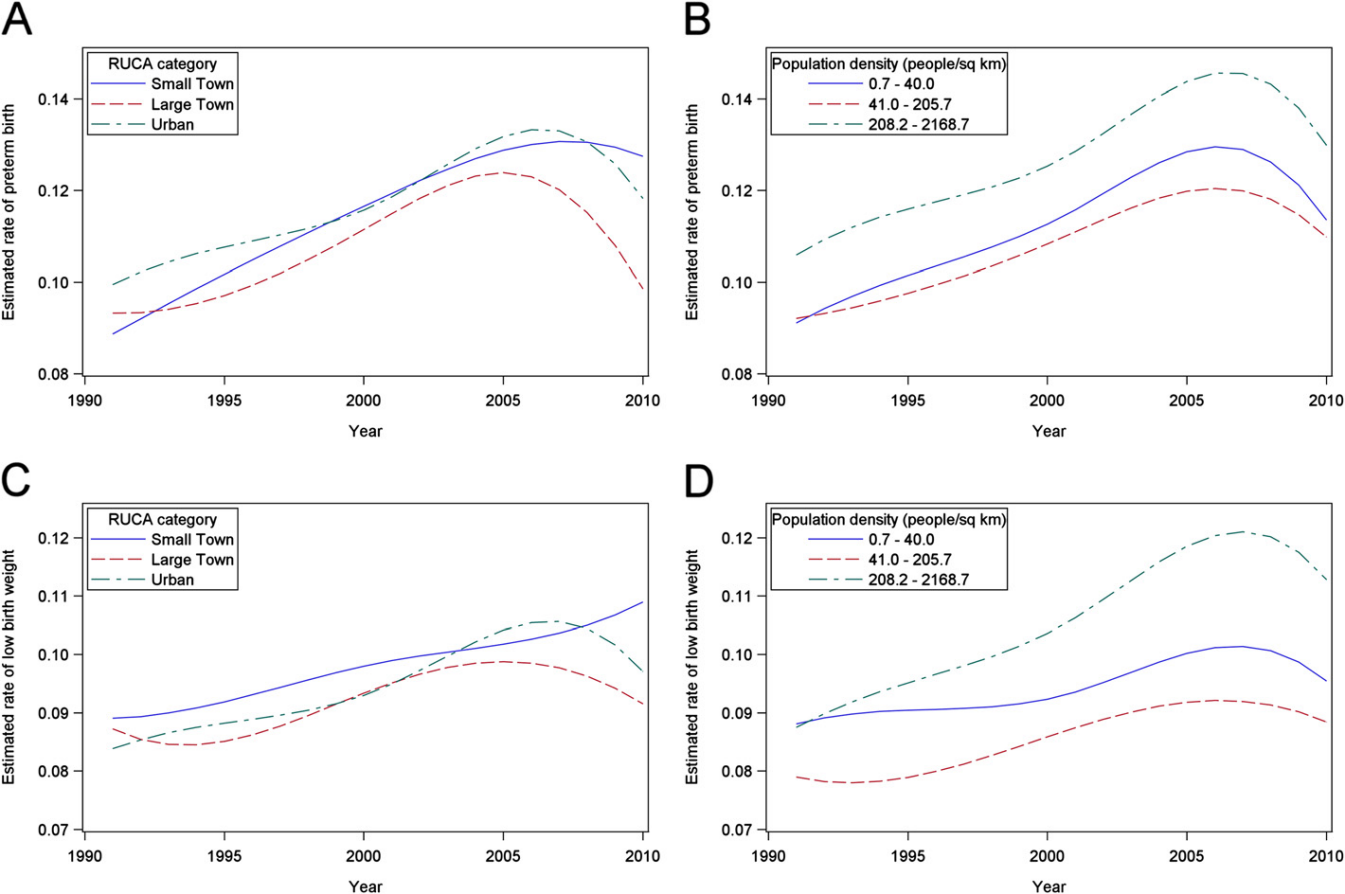
**B-Splines Representing Long**-**Term Alabama Adverse Birth Outcome Trends for (A) PTB by Rural–urban Commuting Area Code category (B) PTB by Population Density Tertile (C) LBW by Rural**–**urban Commuting Area Code category and (D) LBW by Population Density Tertile.**

Figure 2 indicates adverse birth outcome disparities have been widening across urban–rural categories since 1991, despite overall decreasing rates since 2006. At the beginning of the time series (1991), RUCA-defined small town, large town, and urban categories all have similar odds of PTB and LBW babies (Figure 2A, 2C; see Additional file 2). At the peak PTB and LBW rates (2006 and 2005, respectively), small town/isolated and urban area, compared to large town area births, have slightly but statistically significantly higher odds of PTBs and LBWs (Figure 2A, 2C; see Additional file 2). 2010 spline points show the diverging birth outcome patterns by RUCA-defined rurality; large town estimated rates of adverse birth outcomes dropped, but small town/isolated region estimated rates remained higher for both PTB (OR = 1.34; 95% CI: 1.18, 1.52) and LBW (OR = 1.21; 95% CI: 1.06, 1.39) (Figure 2A, 2C; see Additional file 2). There was no evidence of significant pre-peak differences in PTB or LBW birth rate changes (p = 0.20 and p = 0.28, respectively), or evidence of differing post-peak PTB rate changes (p = 0.16) based on year by RUCA interaction models. However, interaction models confirmed post-peak small town/isolated LBW rates continued to rise, while large town LBW rates did not rise and urban LBW rates dropped (p = 0.01).

When looking at PTB trends by population density tertile, the 3rd tertile has PTB odds consistently about 1.2 times the 2nd tertile (Figure 2B; see Additional file 2). Year by population density interaction terms do not support linear PTB rate change differences either pre or post-peak (p = 0.49 and p = 0.24, respectively). However, LBW odds in the 3rd tertile of population density increase more quickly than in the 2nd, showing an OR of 1.12 in 1991 (95% CI: 1.02, 1.23), but an OR of 1.35 in 2005 (95% CI: 1.29, 1.41). This increase in disparity remains through 2010 (OR = 1.31; 95% CI: 1.20, 1.43) (Figure 2D; see Additional file 2). A year by population density interaction model confirmed that pre-peak LBW rates rose faster in higher density ZIP codes (p = 0.0001), and that post-peak there were no linear differences in LBW rate changes (p = 0.84).

When individual-level risk factors were included, RUCA-defined small town/isolated (OR = 1.27; 95% CI: 1.12, 1.45) and urban (OR = 1.16; 95% CI: 1.04, 1.29) ZIP codes still had significantly higher odds of PTB in 2010, relative to large town ZIP codes (see Additional file 3). All significant year by rurality interaction terms remained statistically significant after the addition of individual-level variables (p < 0.05).

### Differing contributions of area and individual level factors to PTB or LBW by rurality

Since ZIP code-level percent poverty and African American stratified by urbanization categories showed high collinearity, they were examined in separate models. Correlation between percent poverty and African American were high in all rurality stratum, but particularly in the more rural stratum (e.g. RUCA-defined small town r = 0.83, P < 0.0001). In addition, the 4th quartile of percent African American in RUCA-defined small town and large town strata had no births in the 1st or 2nd quartile of percent poverty (see Additional file 4). Similarly, all RUCA-defined strata had fewer than 1,000 births in the 1st quartile of percent African American by 4th quartile of percent poverty. Despite having the largest number of total births, the urban stratum had the fewest births (n = 169) in the 4th quartile percent African American by 1st quartile of percent poverty.

Most interactions terms between percent poverty or African American and RUCA in models adjusting for only year were not statistically significant (p < 0.05), so models examining RUCA-specific estimates were not further analyzed. In PTB models, population density significantly interacted with both ZIP code-level poverty and African American in all year-adjusted models (Table 2). Model 1 results (adjusted only for year) indicated that PTB odds had monotonic increases with higher poverty and African American percentages (Table 2). In model 1 results, relationships between ZIP code-level variables and PTB were highest in the highest (3rd tertile) population density category. For example, within the 3rd tertile of population density the 4th quartile of poverty (compared to the 1st quartile) carried an OR = 1.48 (95% CI: 1.34, 1.64), but an OR = 1.08 (95% CI: 0.95, 1.23) in the 2nd tertile of population density. After further adjustment of individual-level variables (model 2), relationships of area-level variables with PTB were greatly attenuated. However, associations between percent poverty and PTB remained significant within the 3rd tertile population density (OR = 1.14; 95% CI: 1.04, 1.24).

**Table 2:** **Odds ratios for associations between ZIP code**-**level percent poverty or African American with 1991–2010 Alabama preterm birth by population density tertile**

	**Population density**		
	**1**^**st **^**tertile**	**2**^**nd **^**tertile**	**3**^**rd **^**tertile**
Model 1: Poverty*population density (interaction P-value = 0.0043)			
1^st^ quartile	1.00	1.00	1.00
2^nd^ quartile	1.09 (0.99, 1.20)	1.00 (0.92, 1.09)	1.18 (1.04, 1.35)
3^rd^ quartile	1.12 (1.02, 1.23)	1.05 (0.95, 1.16)	1.30 (1.13, 1.49)
4^th^ quartile	1.22 (1.11, 1.34)	1.08 (0.95, 1.23)	1.48 (1.34, 1.64)
Model 2: Poverty*population density (interaction P-value = 0.0015)			
1^st^ quartile	1.00	1.00	1.00
2^nd^ quartile	1.09 (1.00, 1.18)	0.96 (0.89, 1.03)	1.07 (0.96, 1.20)
3^rd^ quartile	1.06 (0.98, 1.15)	0.96 (0.88, 1.04)	1.08 (0.97, 1.22)
4^th^ quartile	0.99 (0.91, 1.08)	0.88 (0.78, 0.98)	1.14 (1.04, 1.24)
Model 1: African American*population density (P-value = 0.0084)			
1^st^ quartile	1.00	1.00	1.00
2^nd^ quartile	1.06 (0.99, 1.13)	0.98 (0.91, 1.06)	0.99 (0.83, 1.19)
3^rd^ quartile	1.15 (1.07, 1.23)	1.08 (0.99, 1.18)	1.22 (1.03, 1.45)
4^th^ quartile	1.27 (1.19, 1.36)	1.35 (1.18, 1.56)	1.54 (1.31, 1.81)
Model 2: African American*population density (P-value = 0.071)			
1^st^ quartile	1.00	1.00	1.00
2^nd^ quartile	1.00 (0.95, 1.07)	0.94 (0.88, 1.01)	0.96 (0.81, 1.13)
3^rd^ quartile	1.00 (0.94, 1.07)	0.96 (0.89, 1.05)	1.06 (0.91, 1.24)
4^th^ quartile	0.96 (0.90, 1.03)	1.02 (0.90, 1.17)	1.11 (0.96, 1.29)

Model 1 includes ZIP code-level poverty or African American, population density tertile, and interaction term for the ZIP-level variable and population density tertile, adjusted for year with a B-spline.

Model 2 includes model 1 variables adding parity, payment method, education, race, and a natural spline for mother’s age.

Model 1 relationships between area-level variables carried associations of larger magnitudes with LBW, compared to PTB (Table 3). For example, the relationship between being in the 4th quartile of poverty (compared to the 1st quartile) with LBW carried an OR = 1.79 (95% CI: 1.62, 1.98). However, after further adjustment of individual-level variables in model 2, associations between area-level variables and LBW were similar to associations with PTB. Similar to the results in PTB models, the 4th quartile of poverty (compared to the 1st quartile) was significantly associated with LBW in the highest population density category (OR = 1.12; 95% CI: 1.05, 1.19), whereas percent African American was no longer significant. However it should be noted that in both PTB and LBW models, point estimates for percent African American were similar to percent poverty point estimates (Tables 2 and 3).

**Table 3:** **Odds Ratios for Associations between ZIP code**-**level percent poverty or African American with 1991**–**2010 Alabama low birth weight by population density tertile**

	**Population density**		
	**1**^**st **^**tertile**	**2**^**nd **^**tertile**	**3**^**rd **^**tertile**
Model 1: Poverty*population density (interaction P-value = 0.0043)			
1^st^ quartile	1.00	1.00	1.00
2^nd^ quartile	1.15 (1.04, 1.27)	1.10 (1.01, 1.20)	1.31 (1.15, 1.49)
3^rd^ quartile	1.28 (1.16, 1.41)	1.25 (1.13, 1.37)	1.47 (1.28, 1.68)
4th quartile	1.54 (1.39, 1.70)	1.42 (1.25, 1.62)	1.79 (1.62, 1.98)
Model 2: Poverty*population density (interaction P-value = 0.0010)			
1^st^ quartile	1.00	1.00	1.00
2^nd^ quartile	1.14 (1.05, 1.23)	1.00 (0.94, 1.06)	1.08 (0.99, 1.17)
3^rd^ quartile	1.14 (1.06, 1.23)	1.03 (0.96, 1.09)	1.04 (0.96, 1.13)
4^th^ quartile	1.05 (0.97, 1.13)	0.96 (0.88, 1.05)	1.12 (1.05, 1.19)
Model 1: African American*population density (P-value = 0.0099)			
1^st^ quartile	1.00	1.00	1.00
2^nd^ quartile	1.08 (1.01, 1.15)	1.07 (0.99, 1.15)	1.14 (0.96, 1.37)
3^rd^ quartile	1.26 (1.18, 1.35)	1.27 (1.16, 1.38)	1.51 (1.27, 1.78)
4^th^ quartile	1.56 (1.46, 1.67)	1.64 (1.43, 1.87)	2.06 (1.76, 2.42)
Model 2: African American*population density (P-value = 0.13)			
1^st^ quartile	1.00	1.00	1.00
2^nd^ quartile	0.98 (0.93, 1.04)	0.98 (0.93, 1.04)	1.05 (0.92, 1.21)
3^rd^ quartile	0.97 (0.92, 1.03)	1.00 (0.94, 1.07)	1.12 (0.99, 1.28)
4^th^ quartile	0.94 (0.89, 1.00)	0.98 (0.89, 1.09)	1.13 (1.00, 1.28)

Model 1 includes ZIP code-level poverty or African American, population density tertile, and interaction term for the ZIP-level variable and population density tertile, adjusted for year with a B-spline.

Model 2 includes model 1 variables adding parity, payment method, education, race, and a natural spline for mother’s age.

## Discussion

The first part of our analysis found that RUCA codes defined isolated rural regions whereas population density metrics allowed for examination of highly urbanized areas. We found that long-term birth trends differed by rurality definition. Due to previously increasing national PTB rates, a concerted effort was initiated in 2006 to reduce PTB [1] and overall rates have been declining nationally and in Alabama since then, however our analysis shows adverse birth outcome rates are not decreasing in the most isolated rural regions. LBW rates in particular may still be increasing in these regions. In addition, we found that over the last 20 years, the most urban regions consistently had higher adverse birth outcome rates compared to other regions. In the case of LBW, the disparity between the most population dense and less population dense regions increased during the 1991–2005 time period, and the magnitude of the disparity was maintained through 2010.

Changing demographics and other individual-level characteristics over this time period partially explained the differing time trends, although isolated rural areas maintained elevated adverse birth outcome odds even after these adjustments. Rural regions captured by RUCAs have low commuting flows to metropolitan and micropolitan regions, indicating likely economic and social isolation. Previous literature has found that rural regions have increased prevalence of maternal smoking, and it has been found that lack of social capital is especially tied to maternal smoking in rural regions [12,26]. Rural regions also have more unintended pregnancies and pregnant women in with poor cardiovascular risk factor status than their urban counterparts [13,27]. Finally, maintaining full-time physicians locally, particularly family doctors and obstetricians is more difficult in rural regions [28], and there is increased time-to-care in rural regions [29], so medical advances that have allowed national decreases in adverse birth outcome rates might not be reaching isolated rural regions. The differing time trends by rurality seen in this analysis might be explained if any of these factors changed over time at different rates in isolated rural regions compared to non-rural regions. For example, while smoking prevalence is on a national decline, the prevalence of smoking among pregnant mothers might be decreasing at a faster rate in non-isolated rural regions.

Our analysis found that birth outcome disparities attributable to living in low-income African-American communities were heightened in population dense urban areas, compared to less-dense areas. This result is consistent with a previous study in Pennsylvania, where urban areas had higher preterm birth rates, even after controlling for race and income [20]. Much, but not all of the disparities by ZIP code-level demographic risk factors were explained by individual-level risk factors in our study. Our results may differ from previous studies that found stronger area-level relationships because we have included individual-level factors that account for significant area-level effects in previous studies. In particular, the method of payment may indicate the lack of adequate health insurance, and thus explain adverse birth outcomes associated with reduced access of care in inner city and rural regions [3]. One possible explanation for the remaining effect modification by rurality is that low SES residents in population dense areas have higher environmental burdens that may lead to adverse birth outcomes, such as increased exposure to heavy metals, air pollution, and higher summer temperatures [30-34]. For example, a study in North Carolina found seasonal preterm birth patterns to be more pronounced in urban residents, suggesting urban air pollution may explain the result [35]. Other routes for the effect modification by population density might involve social factors related to adverse birth outcomes, such as inner city crime, stress, racial discrimination, and built environment factors (such as liquor store density and housing damage). These factors may be more prevalent in low SES urban neighborhoods compared to non-urban low SES communities, and might explain the effect modification by rurality [36-39].

Alternatively, it has been previously posited that significant area-level effects may be found not due to causal relationships, but due to structural and residual confounding. Structural confounding may arise from socioeconomic and racial segregation, which in turn may lead to a lack of adequate distributions of individual-level factors within area-level strata [21,40]. This may be addressed by choosing fewer strata (e.g. tertiles versus quartiles), but this in turn may lead to increased residual confounding [21].

There are limitations to the current study. Additional individual-level variables, such as smoking status, alcohol use, psychosocial factors, and disease states of the mothers, would be useful in further describing the relationships we found. Another limitation of this study was that we only had availability to warm-season months (May-September). It is possible that time trends and other relationships investigated differ by warm and cool seasons, limiting the generalizability of our results. Our current analysis does not account for area-level variable changes over the 20 year period. It also has been shown previously that ZIP code-level estimates provide less robust effect estimates than census tract level estimates [41] and that there at spatiotemporal mismatches when census-derived ZIP code tabulation areas (ZCTAs) are merged with postal ZIP codes, as was done in this study [42]. However, ZIP code-level analyses are preferable to larger and even more heterogenous areas, such as counties [13].

## Conclusions

This study finds that adverse birth outcome rates remain higher in isolated rural and more population dense areas, and that these disparities are being maintained or increasing over time. This study also found that relationships between ZIP code-level poverty and adverse birth outcomes were significant in urban areas, even after accounting for individual-level risk factors. Interventions to reduce adverse birth outcomes among low SES populations have been found to be effective, particularly among rural populations, and can be implemented to reduce disparities [43].

## Abbreviations

LBW: Low birth weight; GVF: Green vegetation factor; MODIS: Moderate imaging resolution spectrometer; OR: Odds ratio; PTB: Preterm birth; RUCA: Rural–urban commuting area code; SES: Socioeconomic status; US: United States (of America).

## Competing interests

The authors declare that they have no competing interests.

## Authors’ contributions

STK participated in study design, performed the analysis, and drafted the manuscript. LAM contributed to study design and analysis and manuscript editing. BFZ contributed remote sensing expertise and data and manuscript editing. JMG participated in study design, analysis consultation, environmental health expertise, and helped draft the manuscript. All authors read and approved the final manuscript.

## Pre-publication history

The pre-publication history for this paper can be accessed here:

http://www.biomedcentral.com/1471-2393/13/129/prepub

## Supplementary Material

Additional file 1: Figure S1 Spatial Distributions of (**A**) Percent Poverty and (**B**) Percent African American Quartiles Across Alabama; Spatial Distributions of (**A**) Preterm Birth and (**B**) Low Birth Weight Rates Across Alabama. Click here for file

Additional file 2 Crude B-Spline Odds Ratios and 95% Confidence Interval for Relationships Between Rurality and 1991-2010 Alabama Adverse Birth Outcomes, by Year. Click here for file

Additional file 3 Covariate-Adjusted B-Spline Odds Ratios and 95% Confidence Interval for Relationships Between Rurality and 1991-2010 Alabama Adverse Birth Outcomes, by Year. Click here for file

Additional file 4 Distributions of Alabama ZIP Code Level Percent African American by Percent Poverty Quartiles, Stratified by RUCA 2.0 Defined Rurality. Click here for file
