# Supplementary material for: Area-level risk factors for adverse birth outcomes: trends in urban and rural settings
Source: BMC Pregnancy Childbirth. 2013 Jun 10;13:129. doi: 10.1186/1471-2393-13-129 (PMC3688345; doi:10.1186/1471-2393-13-129)
Supplement: Additional file 1: Figure S1 — Spatial Distributions of (A) Percent Poverty and (B) Percent African American Quartiles Across Alabama; Spatial Distributions of (A) Preterm Birth and (B) Low Birth Weight Rates Across Alabama. [file 1471-2393-13-129-S1.pdf]

Additional Figure 1: Spatial Distributions of (a) Percent Poverty and (b) Percent African American Quartiles Across Alabama

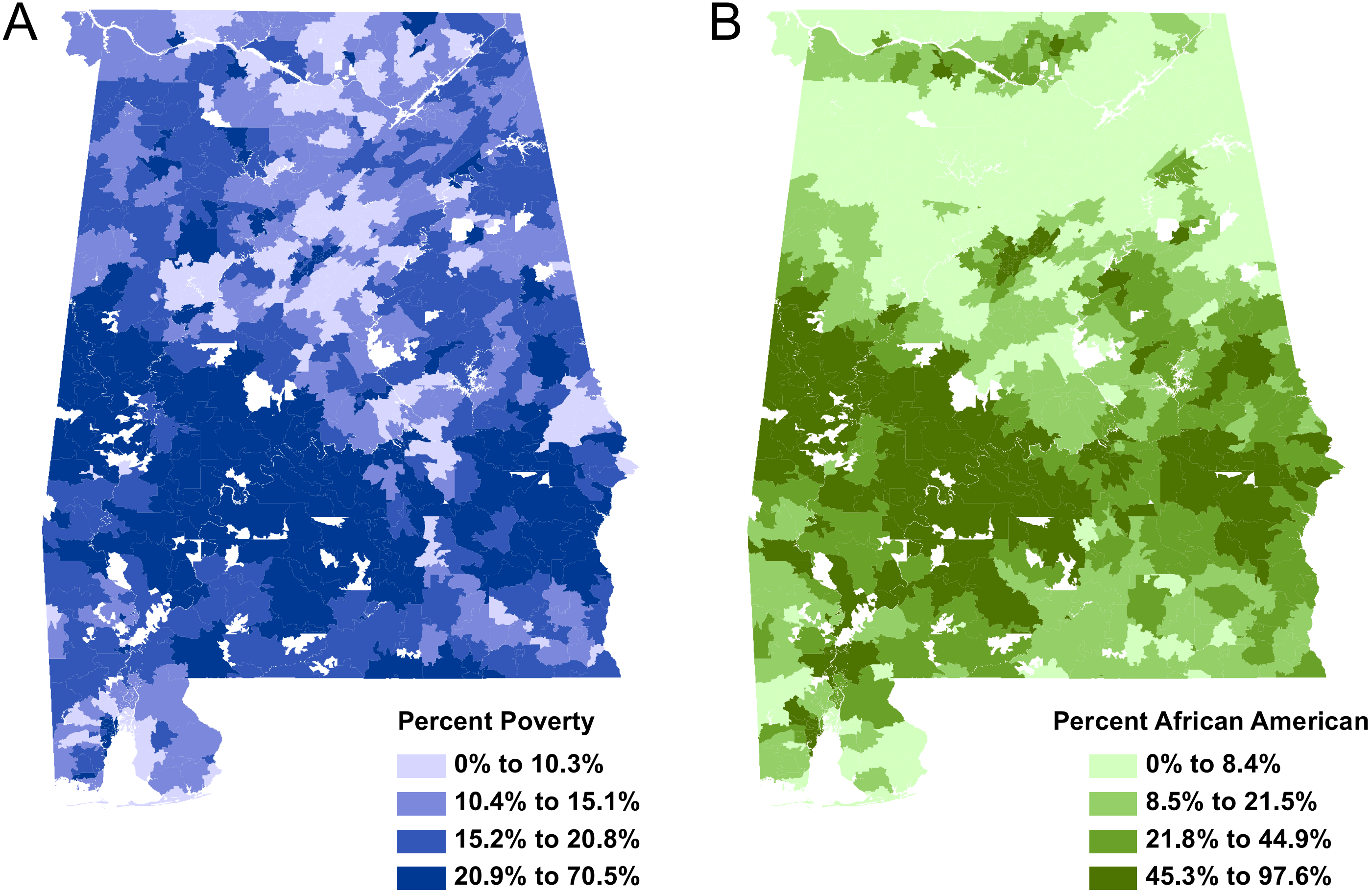
A
B
Percent Poverty
0% to 10.3%
10.4% to 15.1%
15.2% to 20.8%
20.9% to 70.5%
Percent African American
0% to 8.4%
8.5% to 21.5%
21.8% to 44.9%
45.3% to 97.6%

Additional Figure 2: Spatial Distributions of (a) Preterm Birth and (b) Low Birth Weight Rates Across Alabama

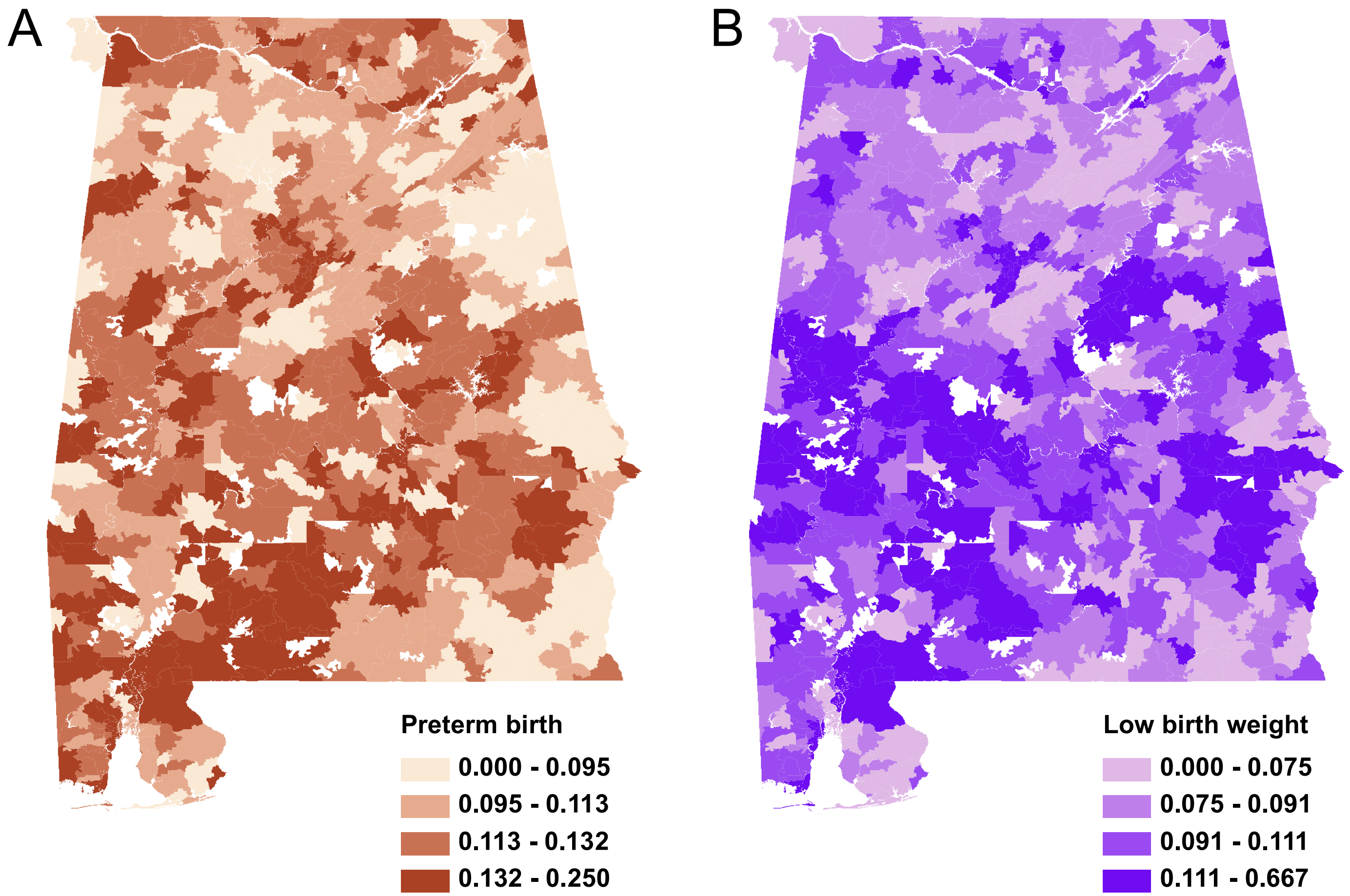
A
B
Preterm birth
0.000 - 0.095
0.095 - 0.113
0.113 - 0.132
0.132 - 0.250
Low birth weight
0.000 - 0.075
0.075 - 0.091
0.091 - 0.111
0.111 - 0.667
